# Guided Slow Continuous Suspension Film Flow for Mass Production of Submicrometer Spherical Particles by Pulsed Laser Melting in Liquid

**DOI:** 10.1038/s41598-018-32528-6

**Published:** 2018-09-21

**Authors:** Yoshie Ishikawa, Naoto Koshizaki

**Affiliations:** 1 Nanomaterials Research Institute, National Institute of Advanced Industrial Science and Technology (AIST), Tsukuba Central 5, 1-1-1 Higashi, Tsukuba, Ibaraki 305-8565 Japan; 2 Graduate School of Engineering, Hokkaido University, Kita 13 Nishi 8, Kita-ku, Sapporo, Hokkaido 060-8628 Japan

## Abstract

Pulsed laser melting in liquid (PLML) is a technique to fabricate submicrometer crystalline spherical particles of various materials by laser irradiation of suspended raw particles with random shapes. To fully exploit the unique features of PLML-fabricated particles (crystalline and spherical) in practice, a mass-production PLML technique is required. To this end, the present study develops a new slit nozzle that guides the suspension film flow into a non-droplet continuous stream with a low flow rate. These two incompatible flow properties (continuity and slowness) are difficult to be realized for a liquid jet to free space. The suspension film flow was irradiated with a typical laboratory scale-flash lamp pumping laser at 30 Hz pulse frequency. Only a single flow passage of the slit nozzle with a few laser pulse irradiation transformed 95% of the raw particles into spherical particles. This spheroidizing ratio exceeded those of low-rate drip flow and high-rate cylindrical laminar flow directly jetted into free space through a Pasteur pipette nozzle. Extrapolating the data obtained from a 20-ml suspension, the average production rate was determined as 195 mg h^−1^. The high spheroidizing ratio and yield through the slit nozzle is attributable to the uniquely slow but continuous liquid film flow. The structure of the slit nozzle also prevents particles from adhering to the slit wall during continuous laser irradiation. Thus, the suspension film flow through the newly developed slit nozzle can potentially scale up the PLML technique to mass production.

## Introduction

The technique of pulsed laser ablation in liquid (PLAL), which generates nanoparticles in laser-irradiated liquids, has been widely studied over the last two decades^1–6^. PLAL generates nanoparticles by ablating the target material with laser irradiation at high energy density. Despite its simplicity and convenience, PLAL is limited in practice by its low mass-production capability. Barcikowski’s group recently achieved a nanoparticle production rate of 90 mg h^−1^ using a laboratory-scale pulse laser with a 10-kHz repetition rate and a pulse energy of 385 μJ^7^. Flowing liquid media and galvanometric scanning of the laser beam can avoid multiple irradiations of the cavitation bubbles in which nanoparticles are generated from the irradiated target surface^8^. Consequently, the beam energy is imparted to the target surface without energy loss by extinction^8^. Adopting this strategy with a high pulse-frequency laser (MHz repetition rate), Streubel *et al*. achieved a production rate of 4 g h^−1^ ^9,10^. Moreover, at production rates above 550 mg h^−1^, gold nano-colloid manufacturing by PLAL is reportedly more economical than the typical wet chemical synthesis method^11^. Thus, laser irradiation onto liquid can potentially realize a practical particle-fabrication technique.

Pulsed laser melting in liquid (PLML) is another type of laser processing technique for submicrometer spherical particle fabrication, in which suspended raw particles are irradiated at relatively low energy density^12,13^. Several pioneering works were reported in the literature for relatively large spherical particle generation by PLAL, although obtained particles were byproducts during nanoparticle fabrication^14,15^ or they are limited only for noble metal and titanium oxide^16–23^. Our group extended this technique to be more powerful by increasing the yield using unfocused laser beam and by developing a method to estimate the size range of produced particle size at specific laser energy density for various materials (not only metals but ceramics and semiconductors)^12,24–27^. To exploit the unique features of particles obtained by PLML (submicrometer size, crystalline, and spherical)^24,28,29^ in practical applications, a mass-production PLML technique is also demanded. In our previous PLML particle synthesis for batch type irradiation, the production rate reached 7 mg h^−1^ under typical conditions^24,25^. To achieve a high spheroidizing ratio of irradiated particles in the vessel, the PLML batch process requires a long irradiation time to compensate the extinction by suspended particles (which limits the effective irradiated space in suspension)^30,31^. Thus, to ensure that all particles suspended in the batch vessel are converted to spherical particles, the vessel is irradiated by an excessive number of laser pulses.

Alternatively, laser irradiation on a suspension of raw particles flowing at an adequate rate should continuously produce submicrometer spherical particles with a high spheroidizing ratio. Barcikowski *et al*. attempted laser fragmentation of dispersed particles in a suspension flow^32^ and extended the concept to PLML^33,34^. A cylindrical suspension flow of 1 mm diameter and a linear flow rate of 60 cm s^−1^ was irradiated with a 100-kHz pulsed laser^34^. Although submicrometer spheres were formed merely by irradiating the liquid flow, a high spheroidizing ratio was achieved only after 50 flow passages^34^. In PLML processes requiring at least 100 mJ cm^−2^ per pulse^24,25,35^, the laser beam must be focused to a diameter of 0.6 mm or smaller, because a typical high-repetition-rate laser delivers at most ~300 μJ per pulse. The mismatch between the beam size and the diameter of the cylindrical suspension flow might explain the multiple flow passage irradiation.

In this study, we irradiate a flow with a high pulse-energy laser (>~100 mJ) for PLML, which is expected to irradiate over a large effective area. Besides a high repetition rate of the laser pulses described above, a large irradiation area should increase the particle mass production. However, the pulse repetition rates of lasers with high pulse energies, such as flash-lamp pumping lasers, are generally low (several–several tens of Hz). Therefore, to avoid non-irradiated through-flow during the intervals between laser pulses, the flow rate for PLML should be slow.

Although the suspension flow rate in a transparent tube is easily controlled, the particles in the irradiated suspension immediately adhere to the inner tube wall, which is fatal for successive mass productions. In contrast, when liquid flow is ejected as a laminar jet from a tube nozzle into free space at a low flow rate, it morphs into a drip flow^36^. Droplet sizes in a drip flow are larger than the diameter of the laminar jet by this morphological change. The long optical length of droplet significantly reduces the laser energy density within the droplet, and hence the spheroidizing ratio.

The present study proposes a new slit nozzle for a suspension film flow, which is suitable for a laser with high pulse energy and a pulse repetition rate of several tens of Hz. After single-flow-passage irradiation of an adequately slow and continuous suspension flow through this new nozzle, high spheroidizing ratio exceeding 90% and high spherical particle yield 195 mg h^−1^ of the product are confirmed using boron nanoparticles as raw particles.

## Results

**Figure 1:**
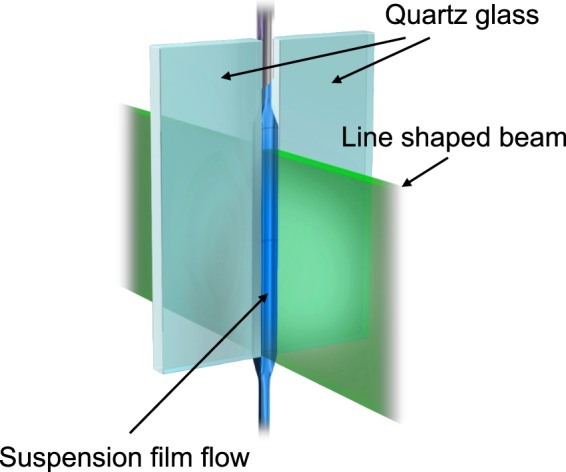
Experimental setup of continuous spherical-particle fabrication by laser irradiation of a guided suspension film flow.

**Figure 2:**
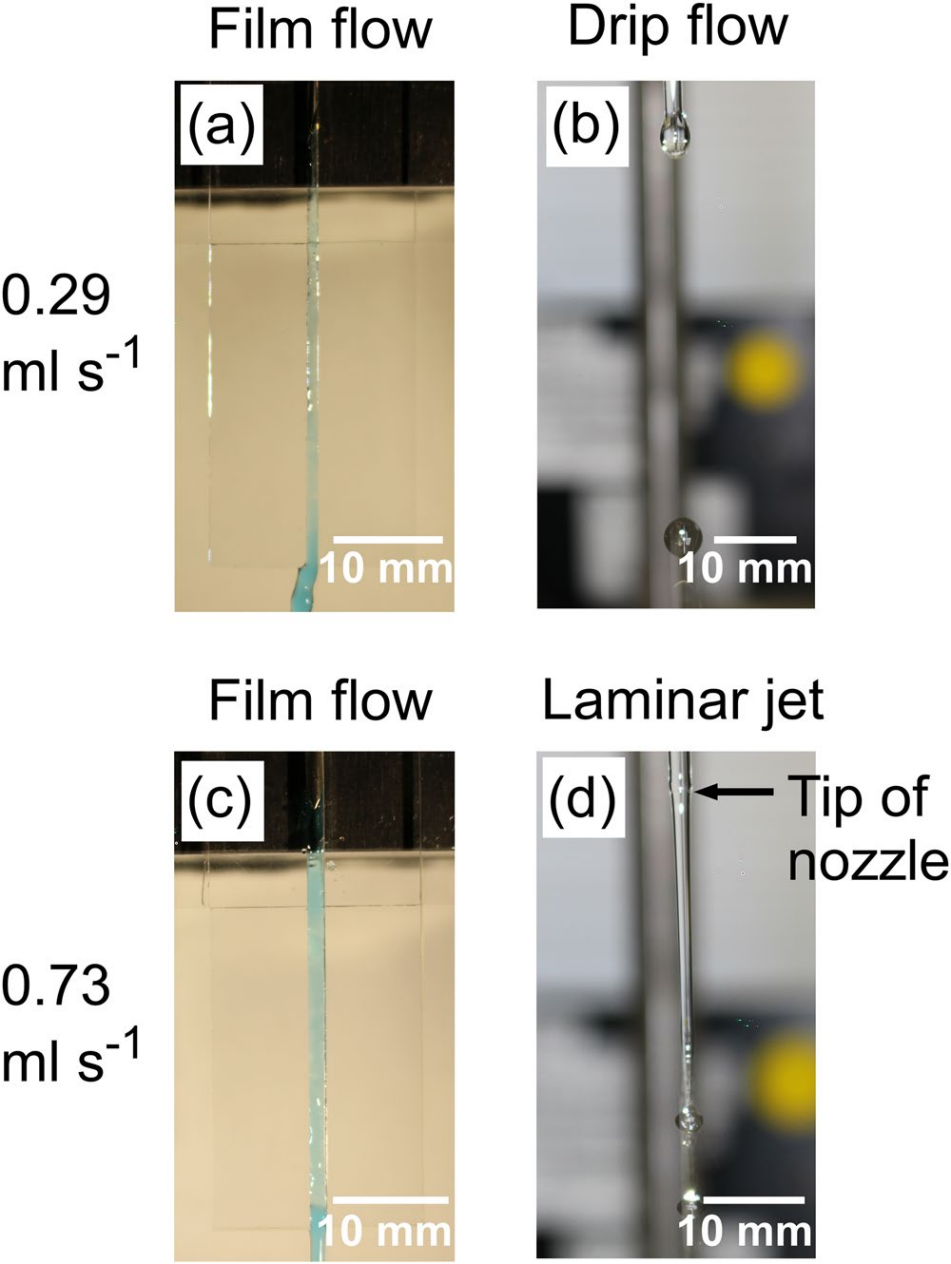
Liquid flows at different volume flow rates (0.29 ml s^−1^ in (**a**,**b**), and 0.73 ml s^−1^ in (**c**,**d**)). The slit nozzle was 1 mm wide and 1 mm thick in (**a**), and 1.5 mm wide and 2 mm thick in (**c**). Flows were visualized by methylene blue aqueous solution. The Pasteur pipette nozzle (inner diameter: 1.4 mm) generated drip flows in (**b**) and a continuous laminar jet in (**d**).

A liquid film flow was guided through a slit-like nozzle, as depicted in Fig. 1. The supplied suspension flowed between the wall surfaces of the slit, forming a film-like liquid flow (Fig. 2(a,c)).

### Flow comparison between the slit nozzle and Pasteur pipette nozzle

**Figure 3:**
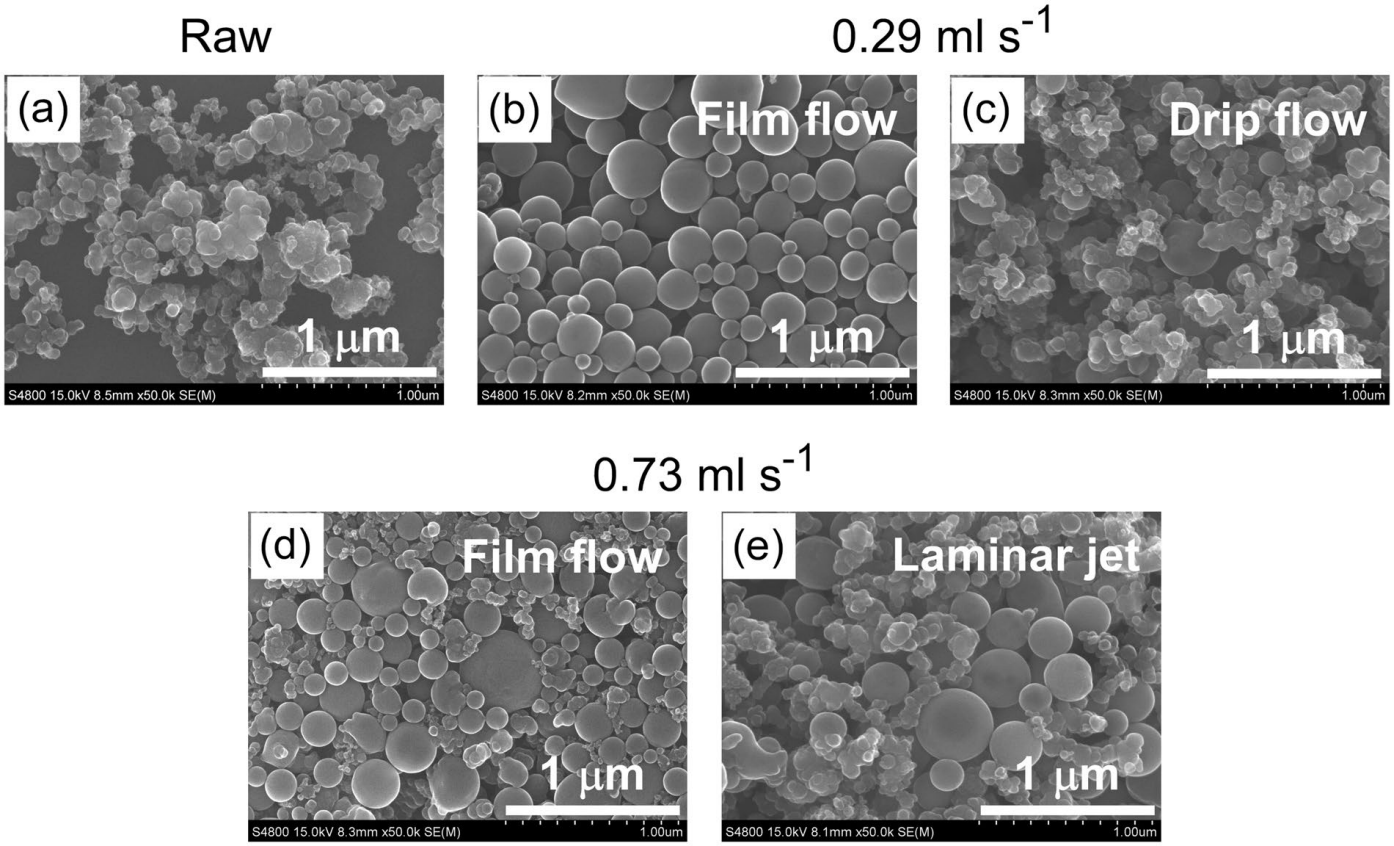
SEM images of boron particles (**a**) before laser irradiation, and (**b–e**) after laser irradiation of boron suspension by single passage of liquid flow. At 0.29 ml s^−1^ volume flow rate, (**b**) through the 1 mm-wide, 1 mm-thick slit nozzle and (**c**) through a Pasteur pipette nozzle in drip flow. At 0.73 ml s^−1^ volume flow rate, (**d**) through the 1.5 mm-wide, 2 mm-thick slit nozzle and (**e**) through a Pasteur pipette nozzle in laminar jet.

The suspension flowed at 0.29 ml s^−1^ along the 1 mm-wide, 1 mm-thick slit (Fig. 2(a)) and through the Pasteur pipette nozzle (Fig. 2(b)). Whereas the flow dripped discretely from the pipette nozzle, the slit nozzle guided the flow into a continuous liquid film. Figure 3 depicts SEM images of non-irradiated boron particles (a), and irradiated boron particles released from the slit nozzle (b) and the pipette nozzle (c). Spherical particles comprised the majority of particles released from the slit nozzle, but a minority of those released from the pipette nozzle.

The flows along the slit and released from the Pasteur pipette nozzle at 0.73 ml s^−1^ volume flow rate are depicted in Fig. 2(c,d). The large slit (1.5 mm wide and 2 mm thick) had to be used at 0.73 ml s^−1^ volume flow rate, because suspension did not stay between the slit and overflowed to the quartz plate surface when the small slit (1 mm wide and 1 mm thick) was used. The slit nozzle released a continuous liquid film as before, but the pipette nozzle released a continuous laminar jet exceeding 30 mm in length. SEM images of the particles obtained through these nozzles are shown in Fig. 3(d,e). The slit nozzle yielded more spherical particles than the pipette nozzle. Thus, the liquid film flow guided by the slit nozzle is more advantageous for spherical particle formation than suspension flow into free space.

The dimensions of width and thickness in slit nozzle will significantly affect not only the flow stability but also spheroidizing ratio and yield. Many further and specific experiments are necessary to estimate quantitatively an effect by the slit nozzle dimension. A discussion of the slit nozzle dimension effect on the spheroidizing ratio and yield was not included in this study, because the aim of this study is to verify the effectivity of film flow irradiation through the slit nozzle.

Suspensions of CuO and Ag nanoparticles were also tested for this slit nozzle (1 mm wide and 1 mm thick) to check the spherical particle formation just by one flow passage. Supplementary Fig. S1 clearly indicates the material versatility of this technique for spherical particle formation. The XRD patterns (see Supplementary Fig. S2) of obtained spherical particles of B, CuO, and Ag with the slit nozzle revealed that crystallinity and crystalline phase of raw particles were retained after irradiation through the slit nozzle. Especially, amorphous spherical particle of boron is exceptionally formed by flow system using slit nozzle as in the case by batch system reported before^12^, since general products by PLML are crystalline. Therefore, particle formation process using the slit nozzle is basically the same as batch process.

### Micro-batch cell irradiation

**Figure 4:**
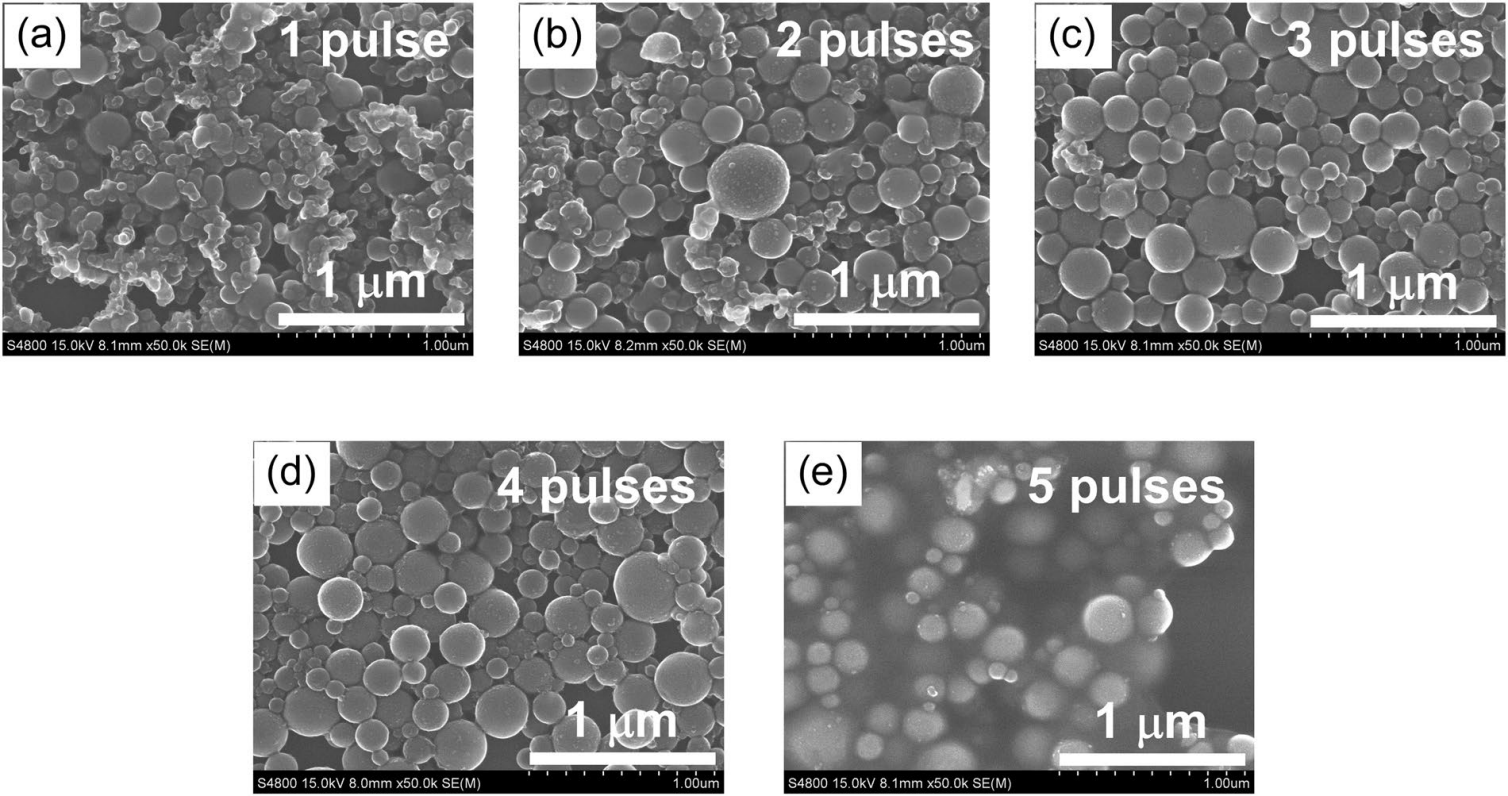
SEM images of the boron particles irradiated with various numbers of pulses in the micro-batch cells.

Figure 4 shows SEM images of the boron particles irradiated with different numbers of pulses in the micro-batch cells. This result shows that a few pulses of irradiation yield spheroidizing ratios of 90% or larger. The spherical particles in Fig. 4(e) are covered with byproduct boric acid formed by repetitive pulse irradiation, revealing that too many pulses irradiation cause oxidation and byproduct formation.

## Discussion

**Figure 5:**
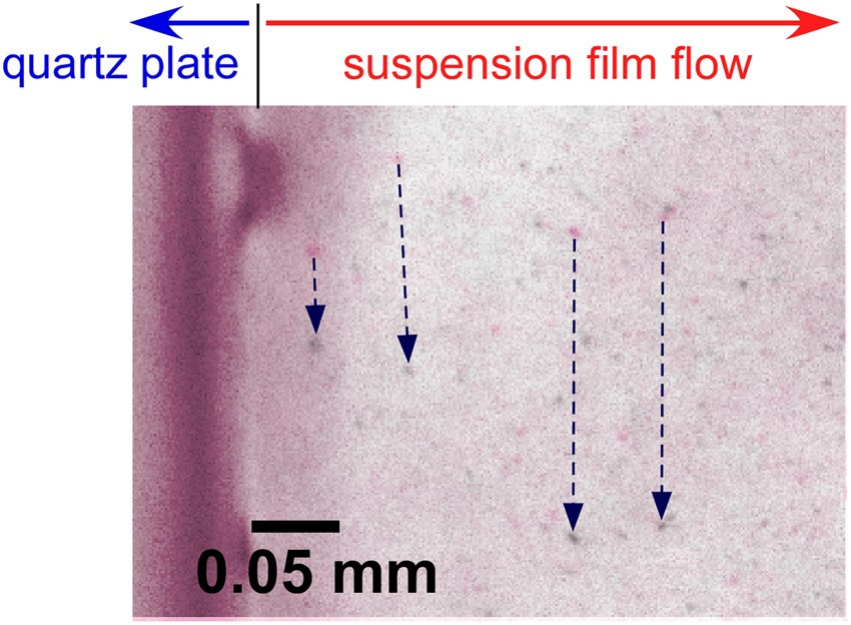
Two superimposed high-speed camera images for analyzing the flow rate of the suspension film. The film flowed through the 1.5 mm-wide, 2 mm-thick slit nozzle. The time lag between the images was 200 μs.

**Figure 6:**
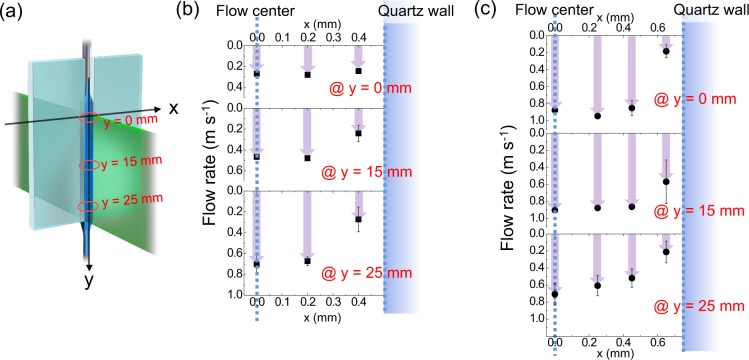
(**a**) Coordinate system of the slit nozzle and flow rate measurement positions along the *y* direction. Linear flow-rate distribution in the suspension film at volume flow rates of (**b**) 0.29 ml s^−1^ and (**c**) 0.73 ml s^−1^.

Figure 5 is a typical high-speed camera image of the flowing suspension film. To analyze the flow rate of this film, we superimposed two images with a time lag of 200 μs, and identified the tracks moved by the particles during the 200 μs (dotted arrows in the figure). By measuring the vertical displacements of these tracks in various parts of the suspension film flow, we evaluated the planar distribution of the film flow rate in the slit nozzle (Fig. 6). In each part of the film, the flow rate was averaged from the results of 10 particles.

Figure 6(a) shows the coordinate system of the slit nozzle. The flow rates (from the flow center at *x* = 0 mm to the quartz wall) were measured at the indicated positions in the *y* direction (*y* = 0 mm, 15 mm and 25 mm). Figure 6(b) plots the flow rates at various positions in the slow suspension film flow (0.29 ml s^−1^) through the 1 mm-wide, 1 mm-thick slit. The *x* values 0.0, 0.2, and 0.4 mm are the central *x* positions in the regions −0.1 < *x* < 0.1 mm, 0.1 ≤ *x* < 0.3 mm, and 0.3 ≤ *x* < 0.5 mm. At each *y* position, the linear flow rate was slowest near the quartz edge (in the region 0.3 ≤ *x* < 0.5 mm) and increased toward the center of the liquid film (−0.1 < x < 0.1 mm). At the center, it reached up to 0.75 m s^−1^ along the *y* direction. From the linear flow rate, the particle retention time in the liquid film flow was roughly estimated as 0.058 s at the center (−0.1 < *x* < 0.1 mm) and 0.10 s at the edge (0.3 ≤ *x* < 0.5 mm). Thus, while traveling through the 1 mm-wide, 1 mm-thick slit, the particles received 1.8–3.1 laser pulses. The high spheroidizing ratio obtained at these irradiated pulse numbers (Fig. 3(b)) accords with the results of the micro-batch cell irradiation, which revealed sufficient spheroidization after a few pulse irradiations.

In contrast, the average time between the beginning of droplet formation and the moment of dropping from the pipette nozzle tip was 0.33 s at a volume flow rate of 0.29 ml s^−1^. Each droplet received at most 10 pulses of the 30 Hz laser. The low spheroidizing ratio (Fig. 3(c)) was mostly caused by optical extinction through the droplet. The optical absorption coefficient of the boron suspension used in this experiment was 0.047 ppm^−1^ cm^−1^. The estimated thickness of the liquid film flowing through the 1 mm-wide, 1 mm-thick slit was 1 mm at most, while the droplets in the drip flow were 3.4 mm thick. In the drip flow, the laser energy density was strongly reduced by the long optical path in the droplet and was insufficient to melt the dispersed particles at low laser energy density side in the droplet. The yield of submicrometer spherical particles in the irradiated suspension droplets might be further reduced by light scattering by the droplets. Thus, for the same volume flow rate and suspension concentration, the liquid film flow yields more spherical particles than the drip flow.

The decrease in laser energy density along the optical path could be suppressed by decreasing the suspension concentration. Although this countermeasure would improve the spheroidizing ratio, it is disadvantageous for mass production because it decreases the throughput per unit time and requires more complex post-processing, such as condensation or particle collection. Thus, the suspension of the thin liquid film guided by the slit nozzle can be prepared at higher concentration than suspension released as droplets into free space.

In the volume flow rate of 0.73 ml s^−1^, the linear flow rate of the laminar jet emitted from the pipette nozzle was estimated as 0.47 m s^−1^ at the nozzle tip. The spheroidizing ratio was clearly higher in the laminar jet from the pipette nozzle (Fig. 3(e)) than in the drip flow (Fig. 3(c)), because the optical length was shorter in the spurted laminar jet (1.3 mm in diameter at 1 cm downward from the pipette nozzle tip) than in the droplet (3.4 mm in thick).

At low and high volume flow rates, the liquid from the tube nozzle was ejected into free space as droplets and a laminar jet, respectively. The threshold volume flow-rate$\,Q$ (cm^3^ s^−1^), at which the flow transitions from droplets to a laminar jet, is obtained as follows^37^:

1 $$Q=1.36\sqrt{\frac{\sigma {D}^{3}}{\rho }(1-\frac{D}{1.24{V}^{1/3}})}$$

Here, $\sigma$ (mN/m) is surface tension of water; $\rho$ (kg m^−3^) is density of water; $D$ (mm) is nozzle diameter; and $V$ (mm^3^) is volume of droplet of drip flow. In the present study using pipette nozzle, a laminar jet is calculated to form, when the volume flow rate exceeded 0.53 ml s^−1^. This threshold accords with the experimental data of Fig. 2(b) (at 0.29 ml s^−1^) and (d) (at 0.73 ml s^−1^). A laminar jet will collapse after travelling a certain distance called the *breakup length*, which is the range used for laser irradiation. The breakup length at any flow rate can be estimated from experimentally determined linear relationship with the flow rate^36^. The minimum breakup length in the pipette nozzle is 0.014 m at 0.53 ml s^−1^, and the breakup length increases with the flow rate. However, the breakup length cannot be longer than 0.14 m (reported value in the nozzle with similar diameter), since the laminar jet transform into a turbulent jet consisting of small droplets^38^. Thus, the permitted laminar jet length in the present pipette nozzle ranged from 0.014 to 0.14 m.

**Figure 7:**
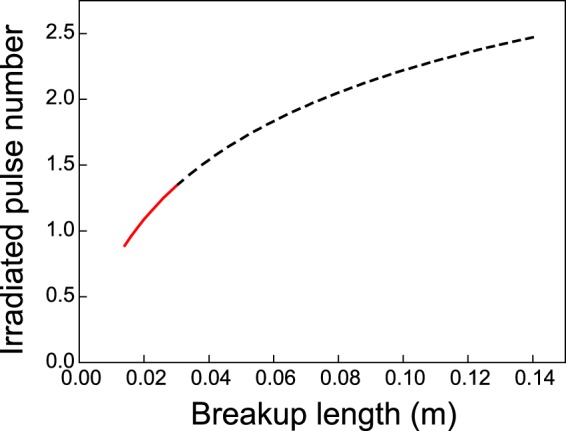
Irradiated pulse number versus breakup length in the Pasteur pipette nozzle. The red solid line indicates the breakup length range permitted by laboratory-scale laser irradiation.

The staying time within a laminar jet of spurted suspension can be estimated from the breakup length, the initial flow rate at the nozzle tip, and the flow rate at the breakup length under gravitational acceleration. Assuming that the spurted laminar jet was wholly irradiated by the line shaped 30-Hz pulsed laser, we calculated the number of irradiated pulses during the staying time at each breakup length. The result is plotted in Fig. 7. The irradiated laminar flow received a maximum of 2.5 pulses within the 0.14-m breakup length. However, sufficient laser energy density for submicrometer spherical particle formation within the 0.14-m breakup length range cannot be provided by the conventional lab-scale pulsed laser. Red solid line in Fig. 7 indicates the range of permitted breakup lengths under irradiation by laboratory-scale lasers (maximum power ~5 W). The laminar jet ejected from the pipette nozzle received only 1.3 irradiated pulses, insufficient for particle formation with a high spheroidizing ratio.

In contrast, the linear flow rate of the 1.5 mm-wide, 2 mm-thick slit nozzle ranged from 0.17 to 0.90 m s^−1^ (Fig. 6(c)), with an average irradiated pulse number of 0.89 to 2.3. The spheroidizing ratio was higher in the slit flow than in the laminar jet released by the pipette nozzle, although part of the rapid film flow received less than 1 pulse. These results are probably attributable to the lower fraction of suspension volume in the fast-flowing regions than in the slow flowing regions, owing to the varying thickness distribution. Near the slit edges (0.55 ≤ *x* < 0.75 mm; see Fig. 6 (c)), where the linear flow rate was slow, the yield of spherical particles was high. At *y* = 25 mm, the flow stagnated because of surface tension at the end sides of the two quartz plates.

**Figure 8:**
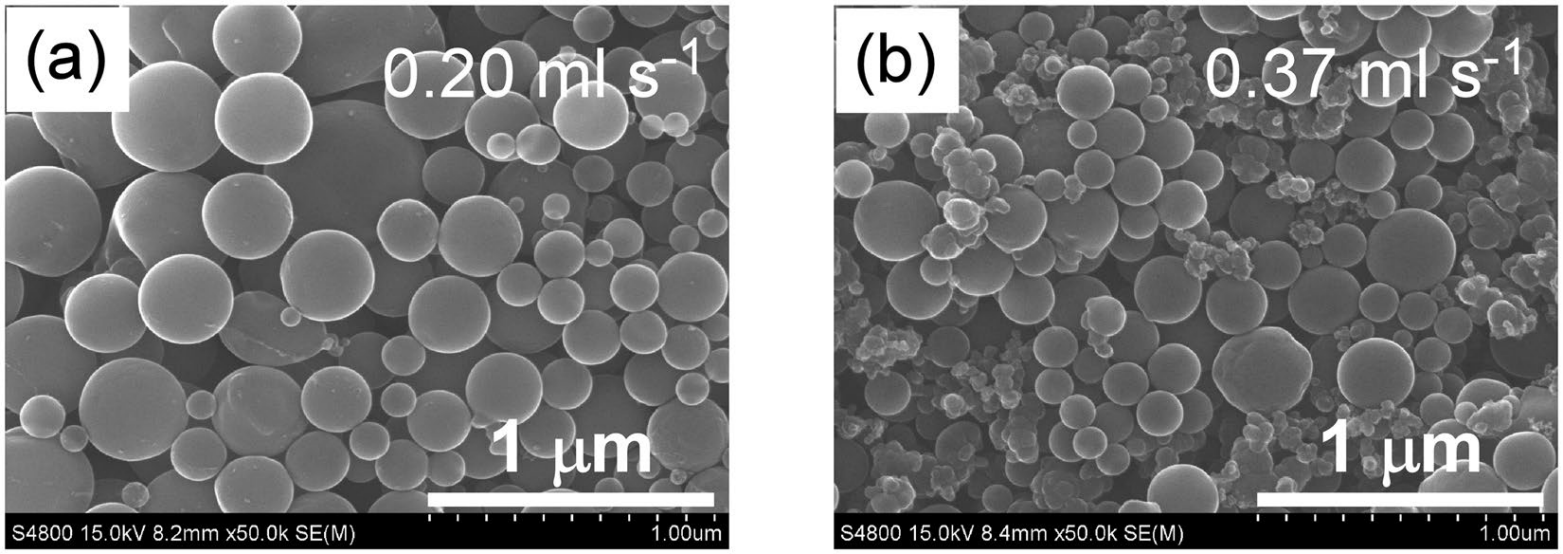
SEM images of boron after irradiation of the liquid film flow by single passage at (**a**) 0.20 ml s^−1^, and (**b**) 0.37 ml s^−1^ through the 1 mm-wide, 1 mm-thick slit nozzle.

**Figure 9:**
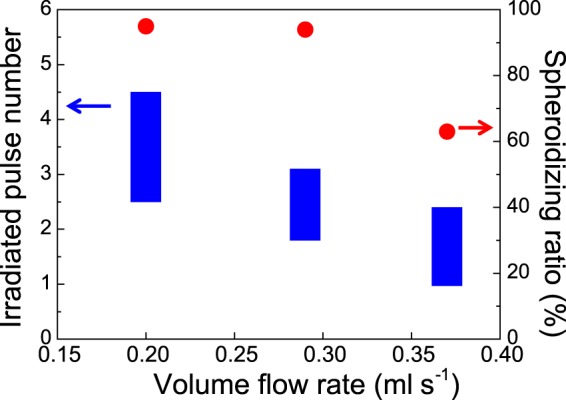
Irradiated pulse numbers (blue bars) and spheroidizing ratios (red dots) at various volume flow rates through the 1 mm-wide, 1 mm-thick slit nozzle.

Figure 8 presents SEM images of the irradiated particles in the liquid film flowing at 0.20 ml s^−1^ and 0.37 ml s^−1^ through the 1 mm-wide, 1 mm-thick slit nozzle. The irradiated pulse numbers and spheroidizing ratios obtained at various volume flow rates are plotted in Fig. 9. The irradiated pulse numbers were higher than the two pulses received by the 0.20 and 0.29 ml s^−1^ volume flow rates. Therefore, the spheroidizing ratios exceeded 90% after a single passage of liquid flow through the slit nozzle. In contrast, only drip flows formed from the pipette nozzle at these low volume flow rates, so the extended optical path seriously reduced the spheroidizing ratio. Thus, the slit nozzle simultaneously achieved two incompatible properties of liquid jetting into free space: low flow rate and non-droplet continuous liquid flow. At 0.37 ml s^−1^, the spheroidizing ratio decreased to 63%, indicating that an excessive flow rate decreases the spheroidizing ratio in the slit nozzle by reducing the number of laser irradiation pulses.

The spherical particle yield (mg h^−1^) was evaluated by extrapolating the data from the 20 ml suspension. The spherical particle yield from the 1 mm-wide, 1 mm-thick slit nozzle was 195 mg h^−1^ at a suspension volume flow rate of 0.29 ml s^−1^. The high spheroidizing ratio and high yield by irradiation on single-passage flow were confirmed by the unique continuous slow flow realized by the slit nozzle. Moreover, the particle adhesion remained minimal even over long continuous irradiation times because the quartz nozzle walls were parallel to the irradiation direction. Thus, laser irradiation of a suspension film flowing through this new slit nozzle is promising for mass production by PLML.

In conclusion, suspension film flows by single passage at various flow rates from a newly designed slit nozzle were irradiated with a typical flash-lamp pumping laser operated at 30 Hz. In the film flow at 0.20 and 0.29 ml s^−1^ under guided by the 1 mm-wide, 1 mm-thick slit, the spheroidizing ratios reached over 90%. Moreover, the spherical particle yield was 195 mg h^−1^ at a suspension volume flow rate of 0.29 ml s^−1^. The spheroidizing ratio of the irradiated particles was higher in the film flow from the slit nozzle than in the free-space flow released from the Pasteur pipette nozzle. The slit nozzle increased the spheroidizing ratio by generating a uniquely slow but continuous flow. Such slow continuous flows are difficult to realize in liquids jetted into free space. The film flow from the slit nozzle is also suitable for continuous irradiation because the slit structure inhibits particle adhesion to the slit walls. Thus, laser irradiation of suspension film flow through the new slit nozzle can potentially realize mass production in PLML.

## Methods

Raw amorphous B nanoparticles (Aldrich, 33,244–5) were dispersed in deionized water at a concentration of 0.2 g l^−1^. Raw CuO (Aldrich, 544868–5 G) and Ag (Aldrich, 576832-5 G) nanoparticles were also used to check the material versatility of this technique. In this study, two slit nozzles were used and depicted in Fig. S3. In the slit nozzle, the top sides of two quartz plates (thickness 1 or 2 mm; length 45 mm) were affixed to either side of a third quartz plate by UV adhesive, maintaining a 1 mm (Slit A) or 1.5 mm (Slit B) gap for the slit. The Slit A (1 mm wide and 1 mm thick) was used for volume flow rates of 0.20, 0.29, and 0.37 ml s^−1^. The Slit B (1.5 mm wide and 2 mm thick) was used for volume flow rate of 0.73 ml s^−1^. The liquid film guided by the 30-mm long slit was entirely irradiated with a line-shaped laser beam. The suspension was fed into the slit by a syringe needle connected to a precision liquid feeder (Furue Science, JP-S). For comparison, we also irradiated the flow from a Pasteur pipette with an inner nozzle diameter of 1.4 mm connected to the precision liquid feeder. By feeding the suspension to the pipette nozzle at 0.29 ml s^−1^ or 0.73 ml s^−1^, we generated drip flow (Fig. 2(b)) and a continuous laminar jet (Fig. 2(d)), respectively. In the drip flow, the droplets (of length 5.0 mm and width 3.4 mm), were released from the pipette nozzle tip at 0.33-s intervals. The flow rate of the particles in liquid film flow was monitored by a high-speed camera (Vision Research, Phantom V2512, shutter interval 100 μs) and measured by image analysis. The laminar flow rate at the pipette nozzle tip was estimated as the volume flow rate divided by the cross-sectional area at the tip.

The flowing liquid was irradiated by a 532-nm Nd:YAG laser with a 30-Hz repetition rate and an 8-ns pulse width (Quanta-ray, Lab-150–30). For irradiating the liquid film flow through the slit nozzle and the continuous laminar flow from the pipette nozzle into free space, the laser beam was shaped into a line beam by cylindrical lenses (see Supplementary Fig. S4). A plane-concave cylindrical lens (*f* = −80 mm), a plane-convex cylindrical lens (*f* = 250 mm), and an optical mask with a 30 mm opening in a vertical direction were placed in order from the laser head. The shaped line beam (2 or 3 mm wide and 30 mm long) was obtained. The drip flow was irradiated just below the tip of the pipette nozzle by the unshaped laser beam (of diameter 8 mm). The average energy density of the laser outputs (both shaped and unshaped) was adjusted to 300 mJ cm^−2^ pulse^−1^. At this irradiation energy density, sufficient spheroidizing was verified from the experimental data of a batch system and from calculations based on thermodynamics and Mie theory^27^.

The required number of laser shots to obtain spherical particles was determined in micro-batch cell experiments. The micro-batches were irradiated from above by a specific number of pulses using a pulse picker (Lasermetrics, 5046SC). The optical transmittance of a 2.5 ppm boron suspension in a micro-batch cell of height 5 mm was 85%. As the inner diameter of the micro-batch cell (4.3 mm) was smaller than the laser beam size (6.2 mm), each laser pulse could wholly irradiate the particles suspended in the micro-batch cell.

The laser-irradiated suspension without any post-chemical treatment was collected and dropped onto a silicon wafer, then dried at room temperature for scanning electron microscopy (SEM) observation (Hitachi S-4800). The spheroidizing ratio was evaluated from SEM images (18 μm^2^) by dividing the total area coverage of the spherical particles by the area coverage of all particles (including raw particles).

For yield estimation of spherical particles, the film flow guided by the slit (1 mm wide, 1 mm thick, and volume flow rate 0.29 ml s^−1^) was used. Totally 20 ml of suspension was irradiated by one flow passage. The produced particles were collected and treated with 0.1 M NaOH aqueous solution to remove the boric acid byproduct, then repeatedly rinsed by centrifugation with deionized water. The rinsed particles were dried and weighed, and the spherical particle yield (mg h^−1^) was extrapolated from the weight of the submicrometer spherical particles obtained by the above procedure.

## Electronic supplementary material

Supplementary Information
