# Supplementary material for: Guided Slow Continuous Suspension Film Flow for Mass Production of Submicrometer Spherical Particles by Pulsed Laser Melting in Liquid
Source: Sci Rep. 2018 Sep 21;8:14208. doi: 10.1038/s41598-018-32528-6 (PMC6155078; doi:10.1038/s41598-018-32528-6)
Supplement: Supplementary file 1 — Supplementary Information [file 41598_2018_32528_MOESM1_ESM.pdf]

Supplementary Information

Guided Slow Continuous Suspension Film Flow for Mass Production of Submicrometer Spherical Particles by Pulsed Laser Melting in Liquid

Yoshie Ishikawa[*,a], and Naoto Koshizaki [b]

[a] Nanomaterials Research Institute, National Institute of Advanced Industrial Science and Technology (AIST), Tsukuba Central 5, 1-1-1 Higashi, Tsukuba, Ibaraki 305-8565, Japan

[b] Graduate School of Engineering, Hokkaido University, Kita 13 Nishi 8, Kita-ku, Sapporo, Hokkaido, 060-8628, Japan

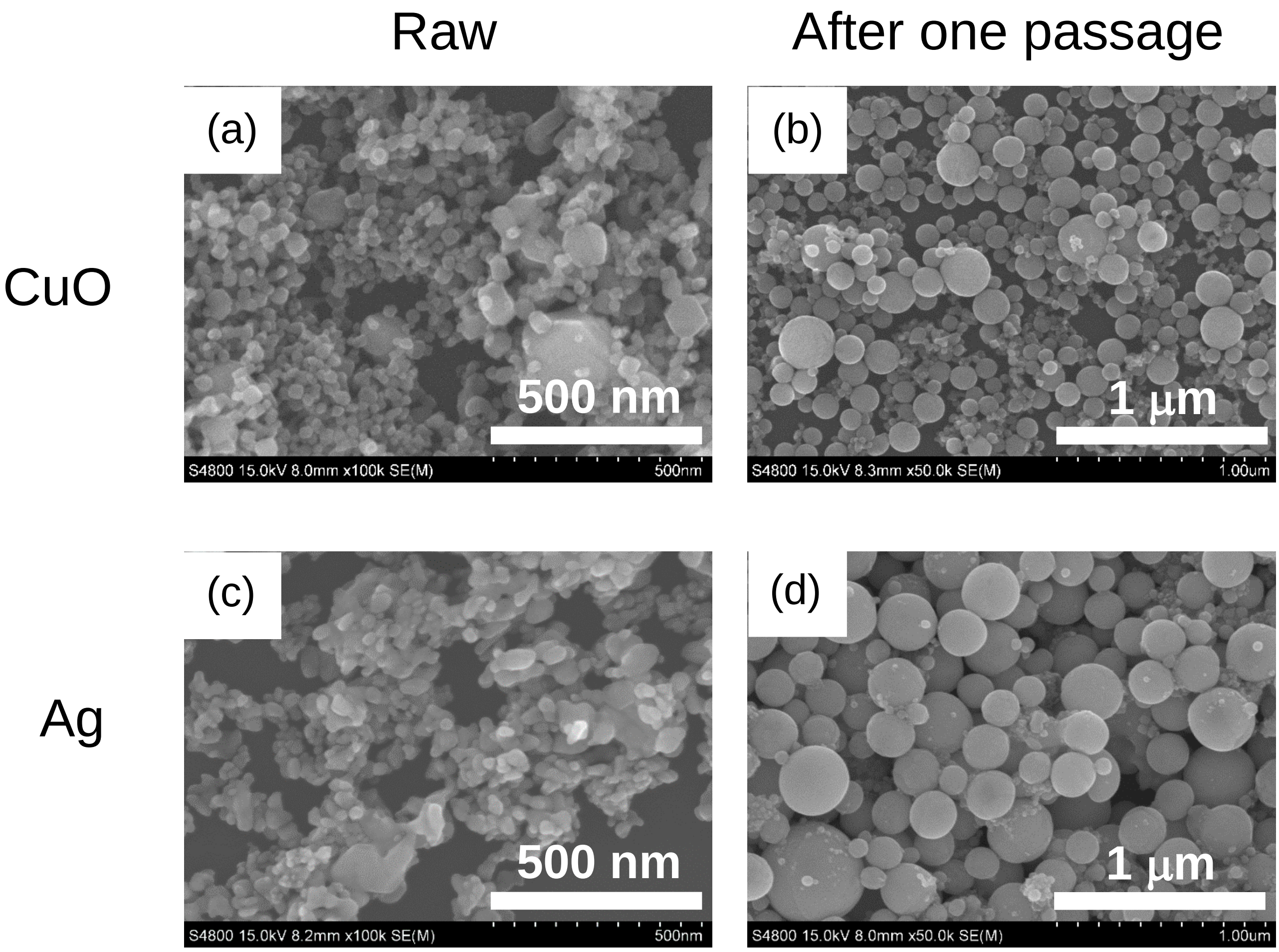

Figure S1. SEM images of raw particles of (a) CuO and (c) Ag before laser irradiation, and those after laser irradiation onto the slit flow (1 mm wide and 1 mm thick) of colloidal aqueous solution of (b) CuO and (d) Ag by one passage at 0.20 ml s^{-1}.

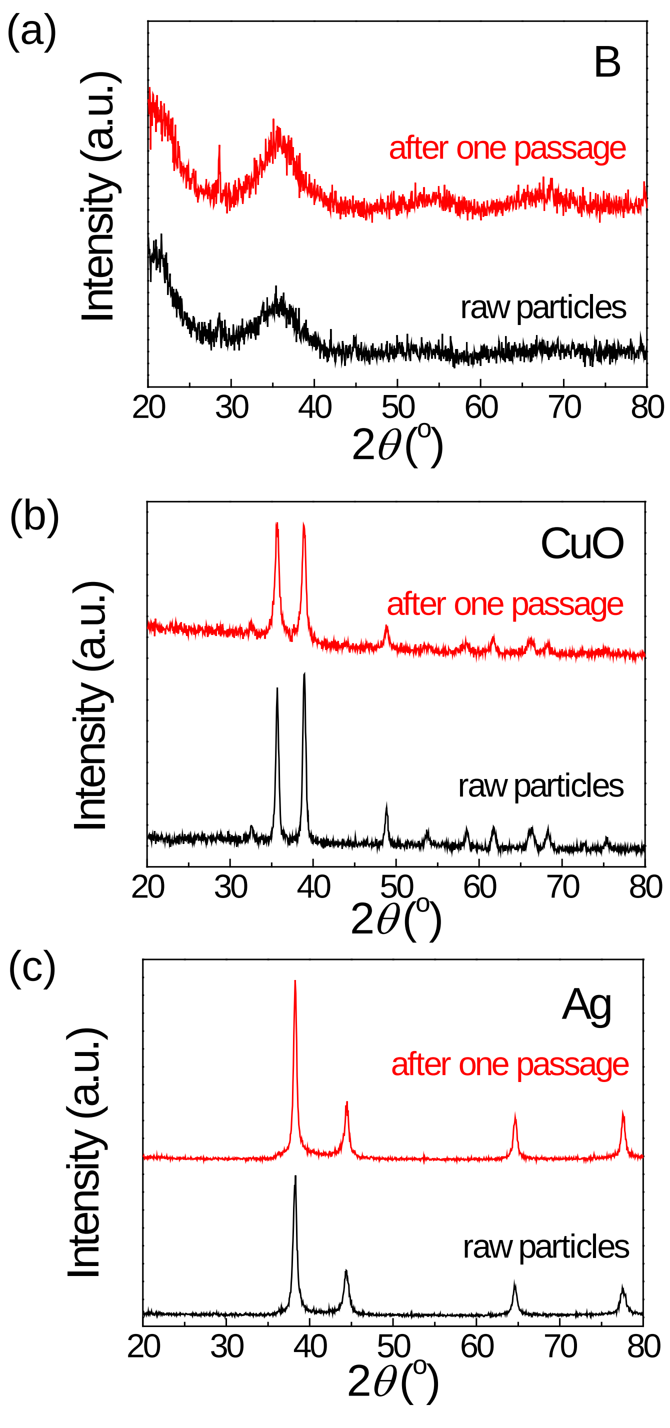

Figure S2. XRD patterns of raw particle and particle after one passage. (a) B, (b) CuO, and (c) Ag.

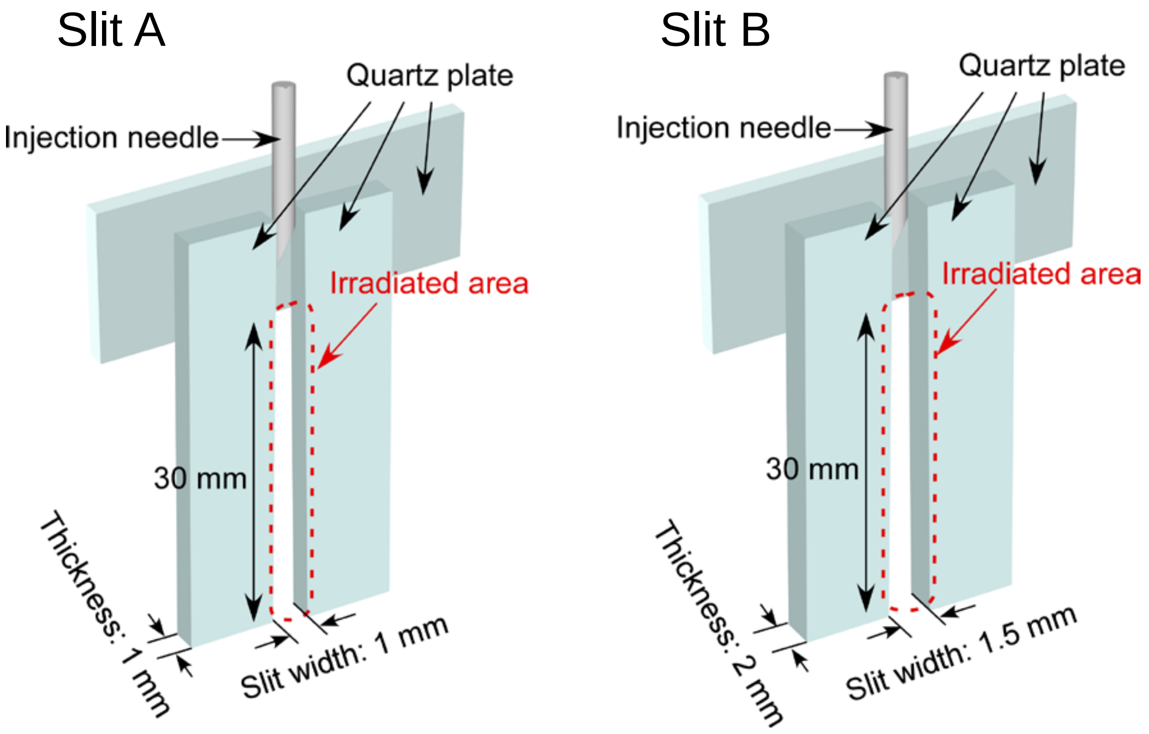

Figure S3. Dimensions of slits used in this study. Slit A (1 mm wide and 1 mm thick) was used for volume flow rates of 0.20, 0.29, and 0.37 ml s^{-1}. Slit B (1.5 mm wide and 2 mm thick) was used for volume flow rate of 0.73 ml s^{-1}.

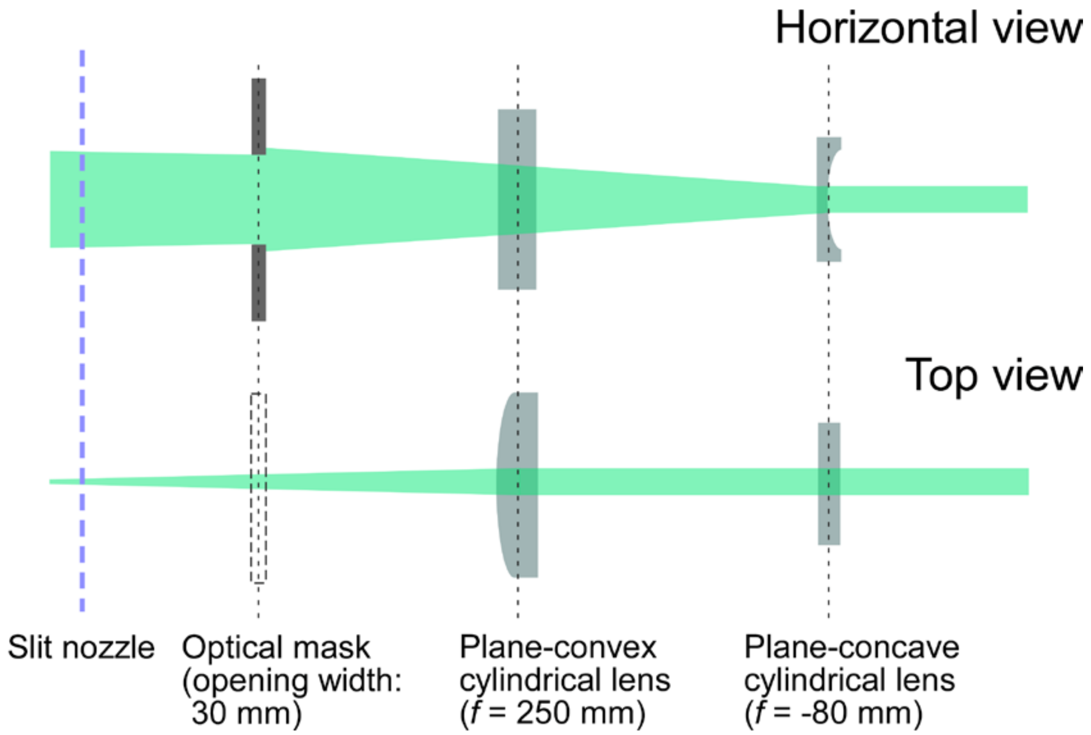

Figure S4. Optical setup for the line shaped beam to irradiate the liquid film flow through the slit nozzle.
